# Magnetization transfer magnetic resonance of human atherosclerotic plaques ex vivo detects areas of high protein density

**DOI:** 10.1186/1532-429X-13-73

**Published:** 2011-11-22

**Authors:** Ye Qiao, Kevin J Hallock, James A Hamilton

**Affiliations:** 1 Department of Physiology and Biophysics, Boston University School of Medicine, 715 Albany Street (W302), Boston, MA 02118-2526, USA; 2 Department of Anatomy and Neurobiology, Boston University School of Medicine, Boston, MA, USA; 3 Department of Biomedical Engineering, Boston University, Boston, MA, USA

## Abstract

**Background:**

Proteins are major plaque components, and their degradation is related to the plaque instability. We sought to assess the feasibility of magnetization transfer (MT) magnetic resonance (MR) for identifying fibrin and collagen in carotid atherosclerotic plaques *ex vivo*.

**Methods:**

Human carotid artery specimens (n = 34) were obtained after resection from patients undergoing endarterectomy. MR was completed within 12 hr after surgery on an 11.7T MR microscope prior to fixation. Two sets of T1W spoiled gradient echo images were acquired with and without the application of a saturation pulse set to 10 kHz off resonance. The magnetization transfer ratio (MTR) was calculated, and the degree of MT contrast was correlated with histology.

**Results:**

MT with appropriate calibration clearly detected regions with high protein density, which showed a higher MTR (thick fibers (collagen type I) (54 ± 8%)) compared to regions with a low amount of protein including lipid (46 ± 8%) (p = 0.05), thin fibers (collagen type III) (11 ± 6%) (p = 0.03), and calcification (6.8 ± 4%) (p = 0.02). Intraplaque hemorrhage (IPH) with different protein density demonstrated different MT effects. Old (rich in protein debris) and recent IPH (rich in fibrin) had a much higher MTR 69 ± 6% and 55 ± 9%, respectively, compared to fresh IPH (rich in intact red blood cells)(9 ± 3%).

**Conclusions:**

MT MR enhances plaque tissue contrast and identifies the protein-rich regions of carotid artery specimens. The additional information from MTR of IPH may provide important insight into the role of IPH on plaque stability, evolution, and the risk for future ischemic events.

## Background

Atherosclerosis is a chronic vascular inflammatory disease that may spontaneously result in stroke, myocardial infarction or intermittent claudication. While considerable research has focused on the complex biochemical, immunological, and signaling aspects of atherosclerotic development [1,2], there is also renewed interest in the ultrastructure of atherosclerotic plaques. Plaques with a lipid-rich core and a thin fibrous cap are likely to rupture, leading to thrombus formation [3]. A stable fibrous cap is mainly composed of collagen derived from smooth muscle cells migrating from the media into the intima. The proteolysis of collagen by metalloproteinases can induce plaque instability by thinning of the cap region. Another common protein-rich component [4] is intraplaque hemorrhage (IPH), resulting from previous disruption of the plaque and/or leaky intraplaque neovessels. The protein composition is different compared to that of the fibrous cap and is mainly comprised of fibrin deposits. Because of the independent roles that each plaque component plays in stability, methods capable of evaluating each specific component (e.g., chemical composition, phase/mobility, and localization) can improve detection of high risk plaques.

Magnetic Resonance (MR) is promising for this goal and has provided complex, information-rich images of atherosclerotic plaques. The most common strategy is to analyze multi-contrast images (e.g., T1 weighted (T1W) and T2 weighted (T2W) and Time-of-flight) that reveal all components with different contrasts [5,6]. Another approach to enhance contrast of a specific component is to develop MR strategies based on differences in molecular mobility and the chemical identity of the component. We have applied protocols *(ex vivo) *that provide high specificity for detection of single plaque component such as calcium phosphate, which can be detected based on its immobility and high concentration of phosphorous by solid state ^31^P imaging [7]. In addition, we exploited the low mobility of protons bound to lipids to improve the contrast between lipids and other components by diffusion weighted imaging (DWI) [8]. We identified these lipids as non-crystalline cholesteryl esters using image-guided ^1^H NMR spectroscopy [9].

In this study we investigated the utility of magnetization transfer contrast (MTC) to detect proteins in carotid endarterectomy (CEA) specimens. MTC is based on a saturation exchange between the protons of macromolecules and water protons when the two pools are coupled by dipolar interactions and/or through chemical exchange. Biological tissues have different sensitivities for magnetization transfer [10] that result in different MR contrast. MT has been used in a wide range of applications, such as soft-tissue suppression in MR angiography (MRA) [11] and delineation of white matter lesions in multiple sclerosis [12]. However, MTC has not been extensively applied to atherosclerotic plaques, and different conclusions have been made as to what components are affected by MT [13-15], raising questions about the reliability of MTR to distinguish components in the complex microenvironment of the plaque. A more pronounced MT effect for the fibrous cap and media compared to the lipid core and adventitia has been reported [13], whereas another group concluded that the regions exhibiting maximal MT effects were associated with areas identified as lipid-rich [14,15].

In this study, our goals were to provide a comprehensive evaluation of MTC at high field (11.7T) applied to CEA specimens and to investigate the feasibility of MT imaging to detect regions with high protein density, such as thick collagen fibers in the plaque and fibrin within IPH. We evaluated the MR results by referencing the histology and addressed the specific question of whether MTC provides tissue-specific contrast beyond that achieved with T1, T2 and DWI MR.

## Methods

Human carotid artery specimens (n = 34) were obtained after resection from patients (mean age = 68 ± 10 years, 24 male, and 19 symptomatic) undergoing endarterectomy with stenosis > 70% by ultrasound, MRA or x-ray angiography. The Institutional Review Board granted the exempt status to this study so informed consent from the patients was not required.

Specimens were rinsed with phosphate buffered saline (PBS, 0.1 mol/L), marked at the proximal end, photographed and transferred to glass tubes containing PBS. Protease inhibitor cocktail (1:100 dilution, Sigma-Aldrich Co, St. Louis, MO) and MMP inhibitors (12 μM, Chemicon Billerica, MA) were added for tissue preservation [16]. The entire preparation process was performed at room temperature in 10 to 20 minutes followed by MR imaging at 37°C for up to 12 hours.

### MR protocol

Images were acquired at 11.7 T on a Bruker Avance spectrometer (Bruker, Billerica, MA) using a 10 or 20 mm (inner diameter) birdcage coil. The temperature was calibrated and controlled at 37°C using a Bruker variable temperature system, which employs an air heater and a thermocouple. The temperature was monitored throughout the experiments, and a sample was equilibrated at the desired temperature for 5-10 minutes when intermittent heating occurred during heavy pulse duty cycles. Serial MT experiments were performed to optimize MTC of plaques. A Gaussian MT saturation pulse was applied with offset frequency of 1.70, 2.89, 4.92, 8.37, 14.2, 24.2, 41.2 and 70 kHz (uniformly distributed along a logarithmic scale), and amplitude (expressed as nutational frequency, ω/2π) of 0.083, 0.167, 0.33, 0.67, 1.34 kHz, respectively. Two sets of T1W RF-spoiled gradient echo (SPGR) images were then acquired with and without the application of a saturation pulse with the optimized parameters (i.e., amplitude of 0.67 kHz, offset of 10 kHz, duration of 12 ms, and time interval between MTC pulse and image sequence of 0.2 ms). The other parameters for the SPGR sequence were TR/TE/Flip angle: 70 ms/5 ms/15°; FOV, 12 mm; slice thickness, 0.5 mm; matrix, 128 × 128; in-plane resolution of 47 × 47 μm^2^, number of average (NSA) of 64, scan time of 33 min and no fat suppression. MT experiments were always acquired within 6 hours of removal prior to other imaging sequences (e.g., T1W, T2W and DWI images).

T2W images were acquired with TR/TE, 3000 ms/40 ms; NSA, 96; resolution, 47 × 47 μm^2^; and a scan time of 60 min. T1W images were acquired with TR/TE, 200 ms/10 ms; NSA, 64; resolution, 47 × 47 μm^2^; and scan time approximately of 60 min. DWI spin-echo images were acquired with TR/TE, 2000 ms/40 ms, attenuator factor (b) of 0 and 1230 sec/mm^2^, respectively.

### Histology

Specimens were fixed in a PBS/10% formalin solution, and embedded in tissue freezing medium (Triangle Biomedical Sciences, Durham, NC) for cryosectioning. Sections (10 μm thick) were collected every 250 μm for each histological method. Hematoxylin-eosin and Masson's Trichrome [5] were used to characterize cellular components, calcification, fibrous tissue, and IPH. Picrosirius red staining (Electron Microscopy Sciences Inc., Hatfield, PA) was used to identify collagen types (type I: yellow/orange and type III: green) under polarized light microscopy (PLM) [17]. Corresponding unstained sections were viewed for PLM to observe the birefringence characteristics of lipids (cholesterol and cholesteryl esters). Immunohistochemistry was performed using the avidin-biotin-peroxidase method (Vector, Burlington, CA). Anti-human mouse monoclonal antibody for Glycophorin A (α) (Sigma-Aldrich, St Louis, MO), a protein exclusive to the erythrocyte membrane, was used to detect IPH.

### Image Registration and Data Analysis

Histological sections were co-registered with MR images as shown previously [8]. MR slices were located by obtaining sagittal view images of the entire specimen and measuring from the proximal end of the specimen or the carotid bifurcation. MR slices and histology were co-registered using distances relative to the carotid bifurcation and internal plaque landmarks clearly visible by MR and histology, including the morphology and size of the lumen and the morphology of large calcified regions. The 15% tissue shrinkage after fixation was considered during the co-registraton [18]. The MR and histology images were matched by scaling, translational, and rotational transforms using ImageJ (National Institutes of Health). Regions of fibrous tissue and lipid identified by histology were used to evaluate the detection of high protein density areas by MR. The signal-to-noise ratio (SNR) of each component was calculated for the three MR techniques (MTC, T1W and T2W) using SNR = (SI_component_-SI_noise_)/SD_noise_, where S_component _was the signal intensity of each component, and the noise was determined within an ROI drawn outside of the specimen. The contrast-to-noise ratio (CNR) of fibrous tissue versus lipid (CNR_fibrous tissue-lipid_), and thick fibers versus thin fibers (CNR_thick fibers-thin fibers_) were calculated as CNR_fibrous tissue-lipid _= SNR_fibrous tissue_-SNR_lipid_, and CNR_thick fibers-thin fibers _= SNR _thick fibers_-SNR_thin fibers_.

MR images were classified into the following groups according to a published AHA Classification criteria [19]: Type IV, atheroma with a confluent extracellular lipid core with possible calcification; Type V, fibroatheroma; Type VI, complex plaque with possible surface defects, hemorrhage, or thrombus, Type VII, fibrocalcific plaques; Type VIII fibrous plaques with abundant collagen and/or SMC-rich lesions without lipid accumulation. The stages of IPH were categorized using previously reported criteria [20]: Fresh, intact red blood cell mixed with fibrin; recent, degrading blood products scattered with remnants of red blood cell membrane; old, amorphous cellular debris with hemosiderin.

MR images were processed with the Paravision 3.0.2 software provided by the vendor. Regions of interest were manually segmented on MR images for each plaque component (e.g., lipid, collagen type I, collagen type III, and IPH (different stages)) based on histological results. MT subtraction maps (MTC) were calculated using the equation: MTC = MT_on_-MT_off_, where MT_on _is the image with MT and MT_off _is the image from the same sequence without a saturation pulse. MTR, which represented signal reduction after MT saturation, was calculated pixel by pixel with the equation: MTR = (MT_off _-MT_on_)*100/MT_off_; and then pseudo-colored as an MTR map using JIM software (Xinapse Systems Ltd, Thorpe Waterville, UK). The PBS on MTR maps was removed using an MTR threshold (MTR < 8%), then the histograms, normalized for plaque areas, were generated. For each histogram, we calculated the peak position (i.e., the MTR value with the highest frequency) and the relative peak height (i.e., the relative voxel counts at the peak position).

Means of the measured MTR values (i.e., lipid, collagen type I, collagen type III, IPH (3 different stages) and calcification) were compared using analysis of variance (ANOVA), followed by pair-wise post-hoc comparisons (SPSS 17.0, Chicago, Ill, USA). Probability values of p < 0.05 were considered significant.

## Results

### Parameter Estimation and Optimization of MT

**Figure 1:**
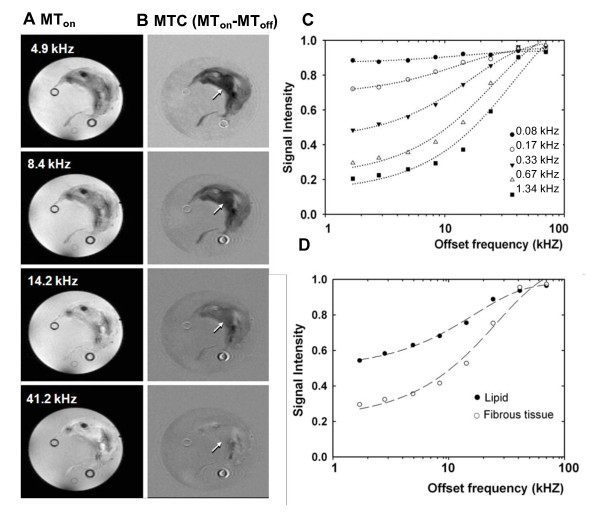
**Optimization of magnetization transfer contrast for a human carotid plaque**. **A**, MT_on_ images acquired with saturation pulse frequency offsets of 4.9, 8.4, 14.2 and 41.2 kHz (logarithmic scale). **B**, The corresponding MT subtraction images (MTC) generated by subtracting the MT_on_ images from the image without MT pulse (MT_off_). Two dark circles (arrows) are glass capillaries to stabilize the specimen. **C**, Normalized SI (SI_s _/SI_0_) plotted versus the offset frequency for five different RF pulse amplitudes (pulse length of 10 ms; region of interest localized in fibrous rich region). **D**, Normalized SI plotted versus the offset frequency for two different components (fibrous tissue and lipid-rich regions) guided by histology (the MT pulse acquired with amplitude of 0.67 kHz and pulse length of 10 ms).

To optimize MT contrast for plaque characterization, MTR parameters were determined from 40 experiments in which the pulse amplitudes of the off-resonance irradiation (ranging from 0.085 kHz to 1.34 kHz) and the frequency offsets (ranging from 1.7 to 70 kHz) were varied. Figure 1A and 1B illustrates MT images acquired with four different offset frequencies on a CEA specimen, respectively. The T1W images with MT saturation (MT_on_) are shown in the left row (Figure 1A) and the subtracted images (MT_on_-MT_off_) in the right row (Figure 1B). The dependence of image contrast on the saturation pulse offset frequency is readily visualized. A small offset frequency (4.9 kHz) results in a strong suppression effect whereas a large offset frequency (41.2 kHz) induces a small saturation effect (i.e., the signal intensity is hardly diminished).

**Figure 2:**
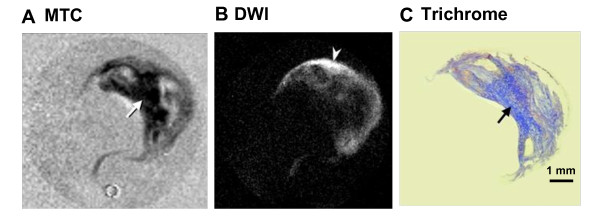
**Correlation of MTC and diffusion weighted imaging (DWI) of a carotid endarterectomy (CEA) specimen with histology**. **A**, MTC. **B**, DWI (b value 1230 sec/mm^2^). **C**, Trichrome. The hypointense signal on MTC (black arrow) corresponds to the blue-stained collagen (collagen type I) on histological image (Trichorme). Lipid deposition is shown as hyperintensity on DWI (white arrow) and accumulates at the peripheral regions of the plaque.

The signal reduction obtained by the combination of different amplitudes and offset frequencies of irradiation pulses is shown in Figure 1C. The signal intensity was measured from dark regions that exhibited maximum MT effects (Figure 1B, arrows). The plot demonstrates that MT saturation effects increased with the amplitude of the irradiation pulses and decreased with the offset frequency. PBS was saturated with an offset below 10 kHz, indicating the direct effect of the irradiation pulse. Therefore, an offset of 10 kHz was chosen to minimize the direct effect. To achieve 50% MT saturation effect, an offset frequency of 10 kHz required the minimum RF amplitude of 0.67 kHz. Figure 1D shows the signal intensity as a function of the offset frequency at fixed amplitude of 0.67 kHz. Lipid- and fibrous-rich regions were determined by histology (Figure 2). ROIs (same as in Figure 1C) were validated as fibrous tissue by the dark blue coloration on trichrome staining. Additional ROIs chosen for the plot of Figure 1D are from the edge of the specimen identified as lipid-rich by DWI (Figure 2B; lipid is bright). This plot demonstrates that the fibrous tissue has a larger MT effect compared with the lipid-rich region and the surrounding saline solution except at very high offset. MT of 60% saturation was observed for the fibrous-rich region and 30% for lipid-rich region with an offset of 10 kHz.

Signal suppression with MT (Figure 2A) was seen in regions that are not lipid-rich, as shown in the corresponding regions of DWI (Figure 2B), the strongest in the region with the highest protein density. The loose connective tissue around the edge of the specimen (seen as gray on MTC) is collagen-poor and overlays the bright regions of DWI, which indicates lipid infiltration within the extracellular matrix (i.e., proteglycan). The results indicate that DWI and MT can create contrast for specific components (lipid and fibrous tissue).

### Magnetization-based Tissue Contrast for Fibrous Tissue Identification

**Figure 3:**
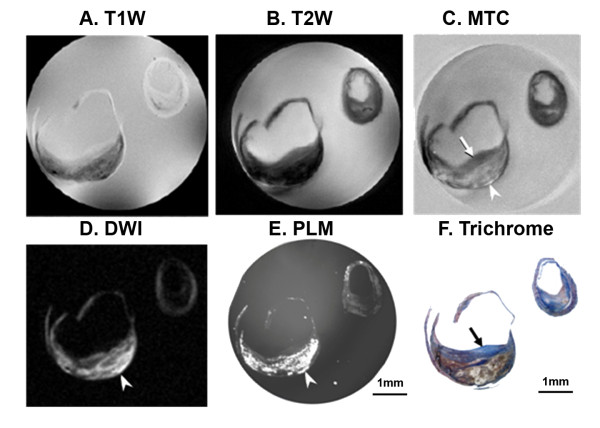
**Muti-contrast MR images of fibroatherma correlated with histology**. **A**, T1W image. **B**, T2W image. **C**, MTC, fibrous tissue is hypointense (arrow) and lipid is hyperintense (arrow head). **D**, DWI image. **E**, Polarized light microscopy (PLM) image of relevant unstained section, and bright signals indicate lipids (arrow head). **F**, Trichrome staining.

**Figure 4:**
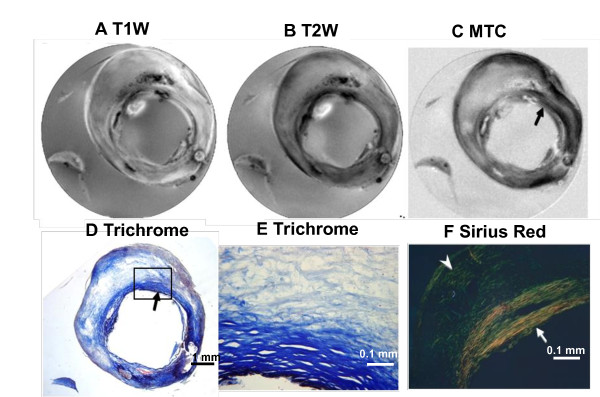
**Muti-contrast MR images of fibrotic plaque correlated with histology**. **A**, T1W image. **B**, T2W image. **C**, MTC, collagen is dark (arrow). **D **and **E**, Trichrome staining. The inset shows a magnified view of collagen type I (dark blue). **F**, Sirius red stain viewed under PLM. Collagen type I is yellow (arrow) and type III is green (arrow head).

**Figure 5:**
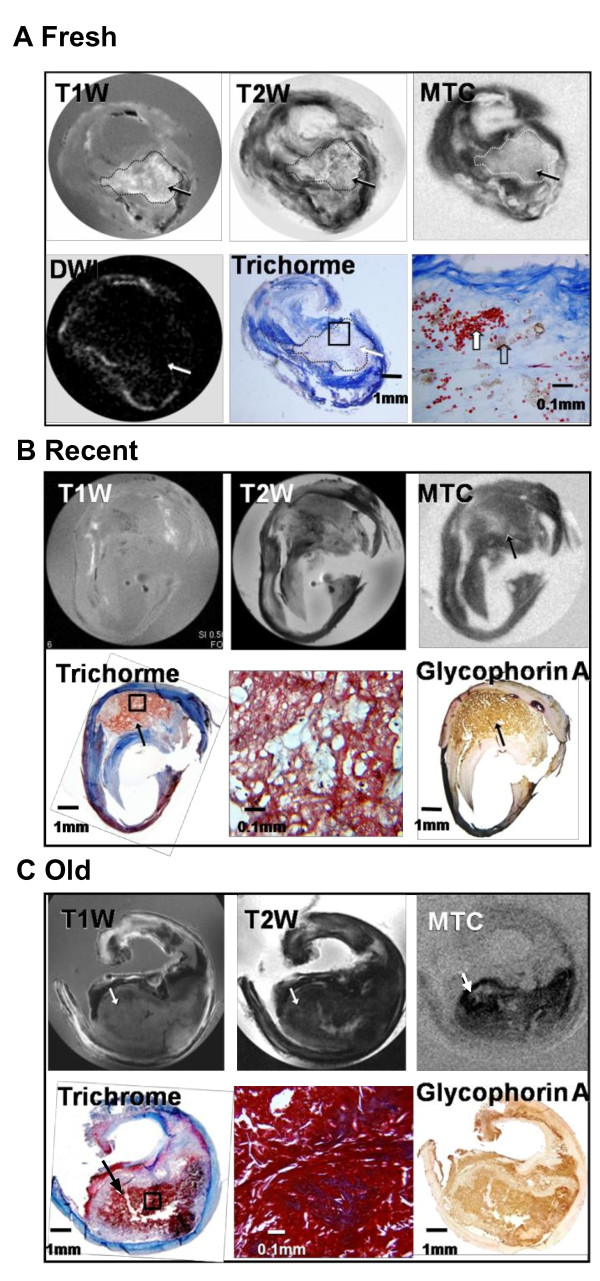
**Muti-contrast MR images (T1W, T2W DWI and MTC) of different stages of intraplaque hemorrhage (IPH) with histology (Trichrome and glyocophorin A) correlation**. **A**, Fresh stage. IPH (arrows) is hyperintense on T1W, isointense on T2W, hyperintense on MTC, and hypointense on DWI corresponding with the red-staining intact erythrocytes on histology (Trichrome). **B**, Recent stage. IPH (arrows) is isointense on T1W, T2W, MTC and hyperintense on DWI. Trichrome shows dense fibrin network (red), and immunostaining for glycophorin A confirms the presence of erythrocytes (dark brown staining). **C**, Old stage. IPH (arrows) is shown isointense on T1W, hypointense on T2W and hypointense on MTC corresponding to the red-staining densely-packed amorphous material. Immunostaining for glycophorin A confirms the presence of erythrocytes (dark brown staining).

**Figure 6:**
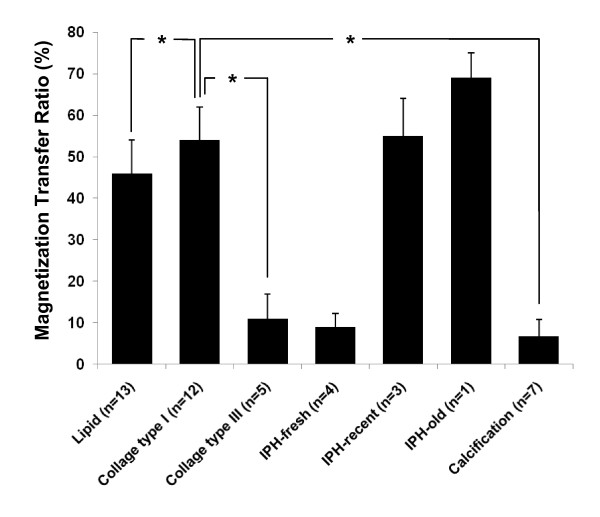
**Magnetization Transfer Ratio (MTR)**. Magnetization Transfer Ratio (MTR) of different plaque components at 11.7T. * indicates statistical significance (p < 0.05) between pair-wise post-hoc comparison using collagen type I as the reference.

Thirty-four CEA specimens were classified according to a modified AHA classification based on histology [19]. The lesions consisted of 12 atheromatous and fibroathermatous plaques (type IV-V), 13 complex lesions with possible surface defects, and IPH (type VI), 7 calcified plaques (type VII), and 2 fibrotic plaques without a lipid core but with possible micro-calcifications. Figures 3, 4, 5 and 6 compare MTC images with other contrast mechanisms (T1W and T2W) for detecting organized proteins in different types of plaques.

Compared with measurements on T1W and T2W images, a difference in SNR measured on MTC for fibrous tissue and lipids could not be detected. However, MTC images had higher CNR _fibrous tissue-lipid _(7.6 ± 2.1) and CNR _thick fibers-thin fibers _(7.0 ± 1.4) compared to T1W (CNR _fibrous tissue-lipid _of 1.3 ± 0.5; CNR _thick fibers-thin fibers _of 0.8 ± 0.5) and T2W (CNR _fibrous tissue-lipid _of 4.5 ± 1.0; CNR _thick fibers-thin fibers _of 4.8 ± 0.5) images.

A common type of carotid plaque is the fibroathermatous plaque (Type V), which contains abundant collagen, a lipid or necrotic core, and sometimes calcification. Figure 3 illustrates corresponding images, above the bifurcation, of a type V carotid plaque in both internal and external carotid arteries (T1W (Figure 3A); T2W (Figure 3B)), MTC (Figure 3C). Signal suppression with MT was seen in regions that are not lipid-rich (arrow, 3C), as shown by comparison with DWI (Figure 3D) and PLM histology (Figure 3E) which both highlight lipids.

Validation of our assignment of the dark regions of Figure 3C to collagen-rich tissue is demonstrated by matched histology (Figure 3F, blue stain with Masson's trichrome). By comparison to the plaques in Figures 1 and 2, (also type V plaques), this plaque has more abundant lipids covered by a fibrous cap, and this microstructure is most clearly revealed in the MTC image.

A less common plaque in our subject population was the fibrotic plaque (2 of 34, Type VIII). This type of plaque usually represents a stable phenotype with abundant collagen and low lipid content, and is not likely to rupture and cause sudden acute events. Figure 4 shows MR images (top panel) and histology (bottom panel) of a fibrotic plaque. The predominant MT effects (dark signal, Figure 4C) occurred in regions identified as collagen-rich by trichrome (Figure 4D and 4E) and Sirius Red staining (Figure 4F). Sirius red staining identifies type I collagen by its characteristic red-yellow color under polarized light. This is the thickest region and is located in the fibrous cap (solid arrow). A large region that shows little MT effect (Figure 4C, within the intima) is rich in type III-collagen, a thinner fiber, which appears green against the black background because of the low density (arrow head, Figure 4F). DW images revealed that the plaque contains very little lipid (data not shown). The T2W image also shows the contrast between the thicker and thinner fibers, whereas T1W is not informative.

### Magnetization-based Tissue Contrast for IPH Detection

The presence of IPH is not only an indicator of plaque rupture [21] but also an implicit indictor for future stroke risk [22]. We investigated Type VI plaques extensively with MT imaging because the IPH can be protein-rich or protein-poor depending on its organization/age, as illustrated by three examples (Figure 5). The MR appearance of this type of plaque was strongly affected not only by the paramagnetic properties of hemoglobin and its metabolites but also by the specific MR contrast method applied.

In a CEA specimen, the fresh stage IPH results in both T1 and T2 relaxation shortening and induces a bright signal in the T1W image and an isointense/hypointense T2W signal (Figure 5A). MTC and DWI did not create contrast for this type of hemorrhage (Figure 5A) suggesting that it has a low content of both protein and lipid. This finding was validated by histology (trichrome stain), which showed a mixture of intact (red stain) and degenerating (brown stain) erythrocytes diffusely dispersed within a loose connective matrix.

A second example illustrates detection by MTC of a recent IPH with a different composition than the previous example (Figure 5B). The large IPH core was iso-intense on both T1W and T2W. MTC gave a relatively high saturation effect, and DWI showed low contrast, which together suggest a protein-rich region with very little lipid. The trichrome stain revealed predominant fibrin strands within the hemorrhage. Immunostain for glycophorin A showed extensive erythrocyte membranes, indicating the presence of hemorrhagic debris.

A third example (Figure 5C) shows an IPH that could not be identified accurately based on the T1W or T2W images but was clearly distinguished by MTC. Histology detected an old (no detectable intact red blood cells) hemorrhage with dense amorphous material (probably cellular debris) and a fibrin mass in its center corresponding to the dark region in the MTC image.

### MTR and Histogram Analysis of Carotid Plaques

MTR values of different plaque components identified in the 34 plaques are shown in Figure 6. The mean MTR was different among plaque components (p = 0.04). The mean MTR for thick fibrous tissue (mainly collagen type I) was 54 ± 9%, versus 11 ± 6% for thin fiber (collagen type III) (p = 0.03), versus 46 ± 8% for lipid (p = 0.05), and 6.8 ± 4% for calcification (p = 0.02). Different ages of IPH demonstrated different MTR; older hemorrhage showed the highest MTR of 69 ± 6% compared with fresh (9 ± 3%) and recent hemorrhage (55 ± 9%).

**Figure 7:**
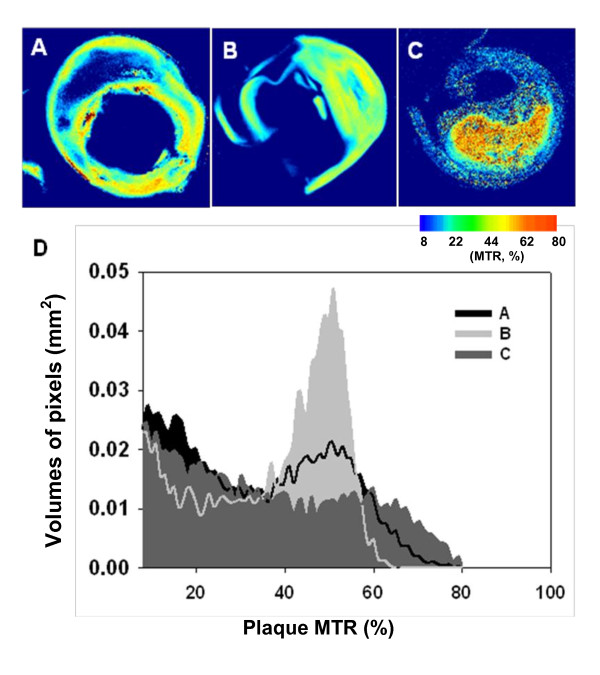
**MTR maps of three CEA specimens**. MTR maps of three CEA specimens **A, B and C**, respectively, with corresponding histograms (**D**). The peak of histograms represents the most common MTR value within each plaque (A B and C correspond the specimen shown in the top panel).

Figure 7 shows the MTR maps (Figure 7A-C) with corresponding group histograms (Figure 7D) for three CEA specimens (A, fibrotic plaque; B; fibroatheroma; C; plaque with IPH, respectively). A higher MTR (≥45%; pseudo-colored as yellow/orange) was observed in regions with type 1 collagen and old IPH (as demonstrated in Figure 4C and 5C as well); MTR of 20-30% (pseudo-colored as green) corresponds to the collagen-poor regions or regions containing the loose collagen type III.

An alternate way to estimate the amount of protein within the plaque is to plot the volume of pixels versus the percentage of MTR (Figure 7D), which however, does not reflect their location within the plaque. The relative amount of each different type of tissue in the plaque can be accessed from the MTR histogram (number of pixels characterized by a certain MTR value). For example, the amount of thick fibers of a specimen B (MTR 40-60%, Figure 7B) is higher than specimen A and C (Figure 7A and 7C), which indicated that specimen B is more fibrotic (rigid) than the other specimens. Thus, the MTR histograms provide additional information to characterize the plaque based on the protein concentration.

The above results indicate that MTC, which originates from differences in macromolecular content rather than T1, T2 and diffusion, allows for differentiation of dense protein rich regions (collagen, especially type I and old IPH) from other plaque components such as lipid-rich region, and loosely packed connective tissue.

## Discussion

In many *in vivo *and *ex vivo *studies, high spatial resolution MR has provided characterization of plaque components and micro-structure, mainly based on molecular relaxation properties (T1 and T2) and proton density protocols. Each of these pulse sequences detects all of the plaque components, and their detailed analysis depends on deconvolution and interpretation of the different signal intensities and contrasts. To focus on protein-rich constituents in plaque, this study utilized MTC to provide tissue specific contrast for the presence of proton exchanging macromolecules, e.g. for collagen fibers. We show that MT with appropriate optimization enhances the detection of organized proteins in plaques. Collagen type I, fibrin and dense protein debris in the carotid plaques showed a high MTR compared to regions with low protein content (lipid-rich and loose connective tissue). Although multicontrast MR has demonstrated the feasibility for differentiating plaque components, we have shown the added value of MTC for generating specific contrast for protein-rich regions (i.e., fibrin, Figure 5C, which was not provided by other contrast mechanisms, such as T2W image).

Fixation of specimens is often used to prevent tissue decomposition, especially with a long *ex viv*o MR acquisition. However, formalin fixation can modify protein structure and further influence the accuracy of MR measurements, such as increasing MT effect and decreasing T2 values [16]. In our study, MR experiments were performed in fresh specimens prior to fixation to avoid the exogenous protein cross-linking induced by formalin.

In addition to discriminating lipids from collagen, MT discriminated collagen type I and type III in carotid plaques. The high concentration of thick fibers (collagen type I) yielded a higher MT effect compared with thin fibers (collagen type III). Presumably these fibers have a relatively large diameter compared to type III fibers [23], and their differences in structure and concentration result in the distinctive MT contrast. Since the macromolecular spins exhibit a much broader absorption line shape than liquid spins, the macromolecular spins can be saturated by off-resonance pre-pulses and transferred to spins of the liquid pool, resulting in a signal reduction compared to a sequence with no off-resonance pre-pulse. The molecular mechanism of MT may originate from the abundant hydroxyl, amine and possibly carboxyl groups in collagen fibers, since these groups play a crucial role for MT [24]. Other mechanisms, such as the hydration layer state, the macromolecule rigidity, and the mobility of hydroxyl groups at the macromolecule surface, might also be involved [25].

In carotid plaques, MT revealed non-uniform distribution of collagen, and dense collagen zones coexisting of loose distributed collagen (e.g. Figure 4D). In fact, a heterogeneous distribution of collagen was better visualized by MTC than the collagen distributed uniformly. We also observed co-localization of the collagen and lipids (e.g., Figure 3A, edge of specimen). Both normal and oxidized LDL can interact with type I and III collagen, suggesting that collagen plays a role in lipoprotein retention during the development of atherosclerosis [1]. It needs to be emphasized that the collagen-rich region does not exclude lipid infiltration. As a plaque continues to develop, collagen concentration may be attenuated with increasing lipid concentration. Our results showing a higher MTR for protein-rich than lipid-rich regions of the plaque is consistent with a previous report describing a pronounced MT effect for fibrous cap (which rich collagen type I) and media, but decreased effects in the lipid core [13]. Those studies were performed on plaque components that were dissected from the arterial wall, and then pooled separately [13,26]. An *ex vivo *application of MT spin-echo at 11.7T to ApoE-/- mice that identified the difference in MTC between fibrous tissue and lipid core showed that MT was relevant to non-human models of atherosclerosis [27].

MR has shown promise in detection of platelet-rich luminal arterial thrombus associated with plaque disruption in both experimental animals [28] and humans [29]. However, MR has not been demonstrated to define the age of the IPH accurately such as identifying old IPH [20]. In our studies we demonstrated that MTC provided additional contrast between IPH and surrounding tissue compared to conventional T1W and T2W MR, and IPH with different histological organization.

**Figure 8:**
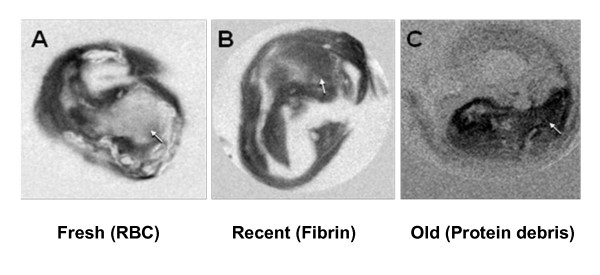
**MTR maps of CEA specimens with different ages of IPH**. MTR maps of CEA specimens with different ages of IPH (**A-C**, arrows). **A**, fresh stage. IPH is with low MTC. **B**, recent stage. IPH is intermediate MTC. **C**, old stage. IPH is with highest MTC.

Finally, to more graphically illustrate both the complexity and utility of applying MTR to advanced plaques with IPH the MTC images of Figure 5 are shown side by side in Figure 8. In a plaque with fresh IPH, as evident by the presence of fresh erythrocytes, the IPH region has very low MTR (Figure 8A) compared to the surrounding fibrous tissue. By comparison, IPH rich in fibrin, which is characteristic of recent IPH, was saturated by MT and had a much higher MTR (Figure 8B). In plaques containing old/organized IPH, which consist of dense proteins, the MT effect was even stronger, creating a very pronounced delineation from the surrounding tissue (Figure 8C). The small MTR observed in fresh IPH with erythrocytes might be due to the strong paramagnetic effects of hemoglobin, which become even more pronounced at high field (11.7 T), and induce a dark signal in T1W gradient echo images. Thick fibers (collagen type I) and fibrin of recent IPH (as established by histology) yielded similar MTR values, suggesting that these two tissue types have the similar organization, concentration and mobilities of their macromolecular pool. The highest MTR associated with old IPH might be due to its highly organized nature and enrichment in cell debris, especially cell membrane proteins, which contribute to the dramatic MT effects [30]. Furthermore, the growth of collagen during the organization of IPH may exert additive MT effects as well [31].

The experiments for this study were performed *ex vivo *at high field (11.7T) which allowed longer acquisition times and a higher resolution compared to most clinical applications, which are at 1.5T or 3T. However, higher field MR is being used with increasing frequency in clinical settings. Because MT is field-dependent, acquisition parameters for individual fields need to be optimized at the chosen field. For this method to become clinically useful, the most important challenge is the spatial resolution (our resolution of ~50 μm at 11.7T) required to detect small pathologic alterations. Currently, the resolution for plaque imaging is about 0.5-1 mm (in-plane) at 1.5T or 3T [19], and it is much lower than the one we proposed, however, with the new developments in MR hardware (e.g., 7T scanner), pulse-sequences and image processing, MTC could be a robust imaging tool that can be readily implemented using existing clinical MR scanners to provide diagnostic potential for complex atherosclerotic plaques and enhance the value of serial imaging. The additional information from MTR of IPHs at different stages may provide a better understanding of the role IPH on plaque stability, evolution, and the risk for future cerebrovascular ischemic events. Determining the age and composition of IPH close to the luminal surface might aid in the selection of therapeutic interventions.

## Conclusions

*Ex vivo *MTC MR enhances plaque tissue contrast and discriminates the protein-rich regions from other components in complex carotid artery specimens, identifies specific types of proteins, and readily detects recent IPH. Future translation to *in vivo *imaging may permit non-invasive identification of plaque components and monitoring of therapeutic interventions for carotid atherosclerosis with the additional valuable information from MTR.

## Competing interests

The authors declare that they have no competing interests.

## Authors' contributions

All authors fulfill the criteria for authorship. JAH conceived the study and handed the funding. YQ and KJH designed MR protocols and carried out MR experiments. YQ performed histology and analyzed data. YQ and JAH wrote the final draft. All authors read and approved the final manuscript.
